# Oral mucosal diseases in anxiety and depression patients: Hospital based observational study from south India

**DOI:** 10.4317/jced.51764

**Published:** 2015-02-01

**Authors:** Kandagal V. Suresh, Prashanth Shenai, Laxmikanth Chatra, Yusuf-Ahammed A. Ronad, Naduvakattu Bilahari, Redder C. Pramod, Sreeja P. Kumar

**Affiliations:** 1 MDS, Senior lecturer, Department of Oral Medicine and Radiology, School of Dental Sciences, Krishna Institute of Medical Sciences, Deemed University, Karad, Satara (District), Maharashtra (State), India; 2 MDS, Senior Professor. Department of Oral Medicine and Radiology, Yenepoya Dental College and Hospital, Yenepoya university, Mangalore (District), Karnataka (State), India; 3 Senior Professor and HOD. Department of Oral Medicine and Radiology, Yenepoya Dental College and Hospital, Yenepoya university, Mangalore (District), Karnataka (State), India; 4 MDS, Senior lecturer. Department of Orthodontics and Dentifacial Orthopaedics, School of Dental Sciences, Krishna Institute of Medical Sciences, Deemed University, Karad, Satara (District), Maharashtra (State), India; 5 MDS, Senior lecturer. Department of Oral Medicine and Radiology, PSM College of Dental Science and Research, Thrisshur (District), Kerala, India; 6 MDS, Senior lecturer. Department of Oral pathology and microbiology, College of Dental Sciences, Davangere, Karanataka (State), India; 37 MDS, Senior lecturer. Department of Oral Medicine and Radiology, Amrita School of Dentistry, Kochi, Kerala (State), India

## Abstract

Objectives: The objective of this study was to evaluate the prevalence of different Oral Mucosal diseases in Anxiety and Depression patients. Material and Methods: A hospital based observational Study was conducted in the department of Psychiatry and department of Oral Medicine and Radiology. Patients who were diagnosed with Anxiety or Depression by the psychiatrists using Hamilton Anxiety and Depression scale were subjected to complete oral examination to check for oral diseases like Oral Lichen Planus (OLP), Recurrent Aphthous Stomatitis (RAS), and Burning Mouth Syndrome (BMS). Equal number of control group subjects were also included. Results: In this study statistically significant increase in the oral diseases in patients with anxiety and depression than the control group was recorded. Oral diseases were significantly higher in anxiety patients (20.86%) than in depression (9.04%) and control group patients (5.17%). In anxiety patients, the prevalence of RAS was 12%, OLP was 5.7%, and BMS was 2.87%. In depression patients, the prevalence of RAS was 4.02%, OLP was 2.01% and BMS was 3.01%. In control group the prevalence was 2.2%, 1.33% and 1.62% in RAS, OLP and BMS respectively. RAS and OLP were significantly higher in the younger age group (18-49) and BMS was seen between the age group of 50-77 years in both study and control groups. Conclusions: The results of the present study showed a positive association between psychological alterations and changes in the oral mucosa, particularly conditions like OLP, RAS and BMS. Thus psychogenic factors like anxiety and depression may act as a risk factor that could influence the initiation and development of oral mucosal diseases. Hence psychological management should be taken into consideration when treating patients with these oral diseases.

** Key words:**Lichen planus, anxiety, depression, burning mouth syndrome, recurrent aphthous stomatitis.

## Introduction

Psychiatric disorders are considerably increasing in last few years and represent a major public health problem. Psychosomatic disorders account for 10% of the global burden of disease, and this is expected to rise to 15% by 2020 (1). Anxiety and Depression are one of the most prevalent psychiatric diseases. These diseases causes physical and pathological changes in the body, oral cavity not being a exception. Oral diseases with psychosomatic etiology have long been identified in medicine but so far these psychosomatic etiologies have not been confirmed. Since oral mucosa is extremely reactive to emotional influences like stress, anxiety and depression; oral diseases may arise as a direct expression of emotions, or indirect result of psychological alterations (2,3). Emotional alterations can disturb hormonal, vascular and muscular functions, which may result in physiologic changes causing pain, burning sensation and ulcerations. Although wide spectrum of psychiatric disorders affects the orofacial region, unfortunately they often are unrecognized because of the common and limited nature of their presenting features (4-6).

Various researchers confirmed that, psychogenic diseases like anxiety and depression causes physiologic changes resulting in the development of oral mucosal diseases like OLP, RAS, BMS. The occurrence of these oral conditions in psychiatric patient has not been studied in a pool of unexplored population of Karnataka or in India as a whole; often the oral health of such patients is undervalued. So, there is increasing need of understanding the distribution of these conditions in patients having psychiatric disorders.

## Material and Methods

The present hospital based observational Study was conducted at the department of Psychiatry and department of Oral Medicine and Radiology in Mangalore over a period of six months. Patients reporting to psychiatry department for the first time, who were diagnosed of Anxiety or Depression by using Hamilton Anxiety and Depression scale by a senior psychiatrist, were subjected to complete oral examination by experienced Oral diagnostician. Equal numbers of controls were included in the study. The controls included were healthy individuals reported to the dental out patient section. Ethical clearance was obtained from the institutional ethical committee. The subjects of all the three groups participated voluntarily, signing a written consent form. Subjects were divided according to age as Adult group [18 - 49 years] and an Older group [50 - 77 years]. The clinical examination of the oral cavity was done following the WHO guidelines, under artificial illumination on a dental chair, using a mouth mirror to check for OLP, RAS and BMS.

Inclusion criteria constituted of the subjects diagnosed with anxiety or depression within the age of 18-77 years. Exclusion criteria constituted of subjects with any systemic diseases, using tobacco; Presence of local irritating factor [sharp cusp, overhanging restoration, calculus. etc]; and subjects under treatment with psychoactive drugs [antidepressants, sedative, narcotics].

The clinical diagnosis of the OLP was established by the presence of a bilaterally symmetrical lacelike gray-white, radiating reticular, annular, plaque-type lesions present at the time of the examination. Clinically diagnosed OLP were subjected to histopathological examination for confirmation.

The diagnosis of RAS was based on the patient’s history and clinical findings. Patients reporting recurrent episodes of round, or ovoid ulcers surrounded by erythematous halo and each episodes of ulceration lasting for a few days to weeks were considered for the study. The ulcers had to be present at the time of the clinical examination.

BMS was recognized when oral burning or pain symptoms, in the absence of detectable mucosal changes were present at the time of clinical examination.

## Results

A total of 278 anxiety and 398 depression subjects and equal number of controls were included in the study. In 278 anxiety patients, 58 [20.86%] had oral diseases and out of 398 depression patients, 36 [9.04%] had oral diseases. Out of 676 individuals in control group, 35 [5.17%] had oral diseases. There was significant increase in oral diseases in anxiety patients [20.86%], depression [9.04%] than control group [5.17%].

Comparison of oral diseases in three groups by using chi square revealed highly statistically significant results between anxiety, depression patients and control group. Comparatively oral lesions are higher in anxiety patients than compared to depression patients and control group patients ( Table 1).

**Table 1:**
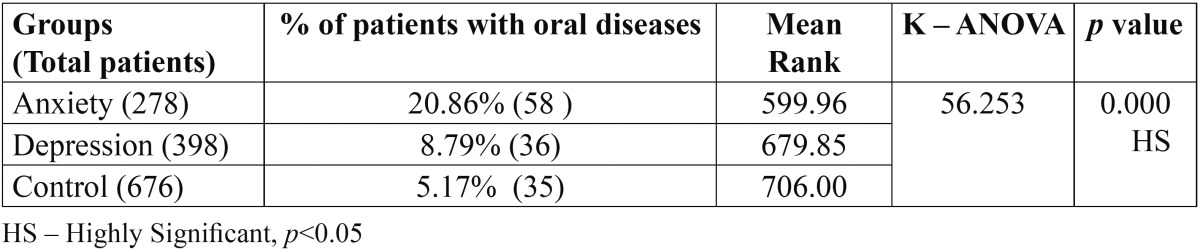
Comparison of oral diseases (overall) among different groups by using chi square.

- Prevalence of oral diseases in anxiety, depression and control group ( Table 2):

**Table 2:**
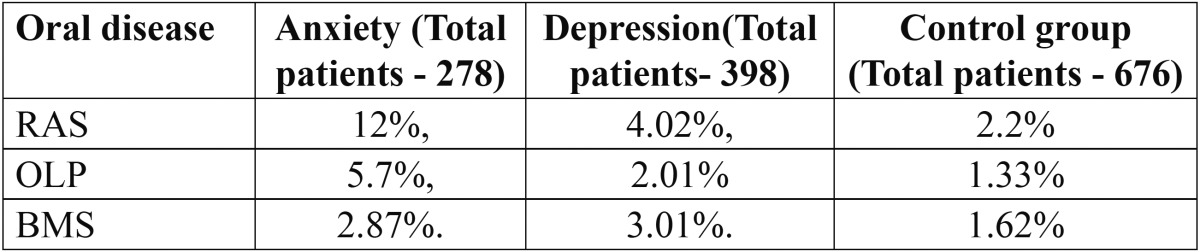
Prevalence of oral diseases in anxiety, depression and control group.

In anxiety subjects, the prevalence of RAS was 12%, OLP was 5.7%, and BMS was 2.87%. In depression subjects, the prevalence of RAS was 4.02%, OLP was 2.01% and BMS was 3.01%. In control group the prevalence of RAS was 2.2%, OLP was 1.33% and BMS was 1.62%.

- Distribution of oral diseases depending on severity anxiety and depression ( Table 3):

**Table 3:**
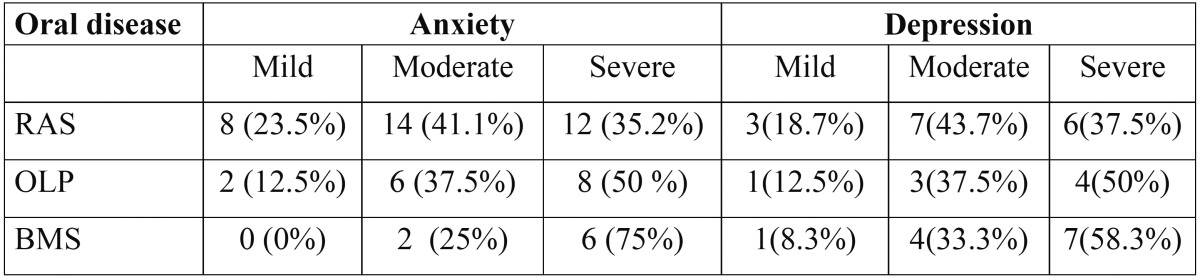
Distribution of oral diseases depending on severity of anxiety and depression.

Patients with moderate and severe anxiety and depression had significantly higher oral diseases than mild anxiety and depression subject.

- Gender wise distribution of oral diseases in anxiety, depression and control group ( Table 4):

**Table 4:**
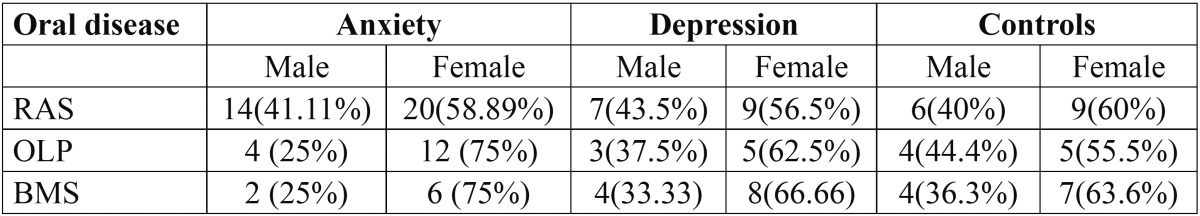
Gender wise distribution of oral diseases in anxiety, depression and control groups.

In all three groups oral mucosal diseases were significantly higher in female patients.

- Age wise distribution of oral diseases in anxiety, depression and control group ( Table 5):

**Table 5:**
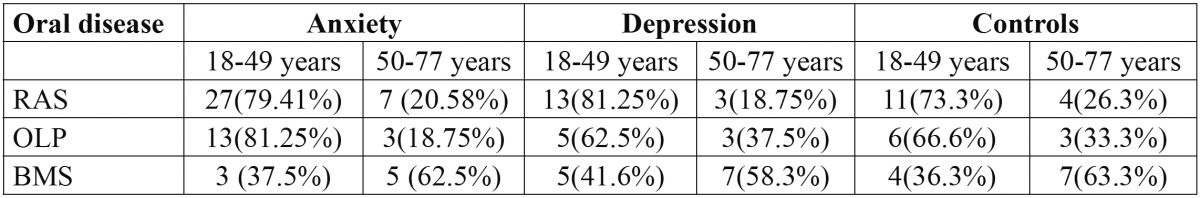
Age wise distribution different oral diseases in anxiety, depression and control groups.

In all the three groups, RAS and OLP were significantly seen between the age group of 18 - 49 years, and BMS is seen between the age group of 50-77 years.

Besides these three oral diseases, majority of the psychiatric patients also showed other oral diseases like Xerostomia, Herpes labialis, Myofasial pain dysfunction syndrome, Geographic tongue, Fissured tongue, Dysguesia, Bruxism, Atypical facial pain and Lip or Cheek bite, in order of decreasing prevalence.

## Discussion

Emotional factors have potential influence on the body; it causes pathological changes or subjective symptoms in normal oral mucosa. Several studies have attempted to elucidate the possible role of psychological state, emotional instability and personality modulation in precipitation of various oral diseases like RAS, OLP and BMS, but no studies are available on the prevalence of oral diseases in psychiatric conditions like Anxiety and Depression (2,4). It is proposed that psychological disturbances procreate the development and worsening of the oral diseases. Many researchers found that oral diseases frequently undergo periods of remissions and exacerbations that often clearly relate to the patients emotional status (4). Since the oral tissues are highly reactive to psychological influences, oral symptoms are common psychosomatic manifestation. Psychological factors results in the alteration in the nervous system markers [Catecholamines; Adrenaline, Noradrenaline, and Dopamine], Endocrine system markers [Cortical and Aldosterone], and Immune system [T cells, B cells and Natural Killer cells, Immunoglobulin’s] resulting in the initiation/ pathogenesis of the oral disease (6). No previous studies are available to compare the prevalence of oral diseases in psychological altered conditions like anxiety and depression. However many studies have evaluated the prevalence rate in general population. The difference in prevalence rates could be attributed to different demographic variables, genetic factors and difference between the racial groups. Many researchers evaluated the stress, anxiety and depression levels in patients suffering from oral diseases. They concluded that significantly higher stress, anxiety and depression levels were found in the RAS, BMS and OLP patients when compared to controls (3,4,7,8). However many studies have assessed the prevalence of OLP in general population, in contrast, the present study evaluated the oral mucosal diseases in anxiety and depression patients which could explain higher prevalence of OLP in present study.

Suwarna Dangore-Khasbage et al. evaluated the prevalence of RAS, BMS, and OLP in institutionalized and non-institutionalized psychiatric patients, They found that the prevalence of RAS, BMS, and OLP was 19.33%, 20.66% and 5.3%, respectively, in all psychiatric patients (1). In the present study prevalence rate was lesser because we considered only new anxiety and depression patients not under any previous medication. Authors also reports that the high prevalence of RAS, BMS and OLP in psychiatrist patients, can be due to increased psychological stress and psychiatric illness can modify immunological functions (9). Psychological investigations have reported that the oral mucosa is a complex and vulnerable region that is very reactive to certain psychological influences. Hence, on the basis of the available literature, the reasons for increased prevalence of RAS, BMS and OLP in psychiatric patients may be multiple and involve the interaction of biological and psychological systems (6).

Results of the present study are comparable to the findings of the previous studies in relation to the prevalence of oral diseases in general population.

B. E. McCartan *et al.* conducted comprehensive reviews of OLP in the period 1980-2007. An overall age standardized prevalence of 1.27% [0.96% in men and 1.57% in women] was calculated from the study. They concluded that furthermore, well-designed studies, with agreed methodology and criteria, are required in a number of different populations for drawing strong conclusions on the prevalence of this condition (10). A more recent Indian study by Saraswathi T *et al*, reported a prevalence of 0.15% of OLP (11). Another latest survey by Mathew AL *et al.* reported a prevalence of 1.2% in Indian population (8). Many previous studies reported prevalence of 1 to 2% in general population (5,7). The prevalence rate of OLP in our study was found to be higher than reported by, the above mentioned researchers.

In the present study, the prevalence of RAS was 12% in anxiety and 4.02% in depression patients, and 2.2% in control group suggesting a higher prevalence rate than that reported in the previous studies by Mathew *et al.* in Indian normal population [2.01%], (8), Rivera-Hidalgo F *et al.* [0.89%] (12), Chattopadhyay in United States (13). In a cross-sectional study by Mumcu G in Turkey population, RAS were observed in 1.2% of the examined patients (14). This is consistent with the results of the present study. This could be explained by that psychiatric subjects might be different than other subjects in term of emotional alterations, stress, lifestyle and other related factors.

In regard to RAS, prevalence rate observed in the present study was lower than that reported by the previous studies. The occurrence of RAS in general population ranges between 5% and 20% (5). Recent survey by Davatchi *et al.* in Tehran revealed a prevalence rate of 25.2% (15). Szponar E *et al.* in their 10-year retrospective observations in Iran reported a prevalence rate of 7.6% (16). A latest survey from Jordanian population reported a prevalence of about 78% of subjects diagnosed with RAS (17).

The severity and frequency of the episodes vary on a case-by-case basis; however, it usually decreases with age. Many epidemiologic studies and our own observations confirmed the higher incidence of RAS in people with higher psychological alterations.

The recent study on the prevalence of RAS showed that the occurrence of RAS varies with the patient population depending on their ethnic origin and the diagnostic criteria system accepted in different research centers. The actual prevalence rate of RAS is greater than the reported rates because of the recurrent nature of the condition and cross-sectional clinical surveys might probably underestimate the true prevalence rate because active lesions may not be present at the time of examination (5,7).

The prevalence of BMS in anxiety patients were 2.87%; while depression patients showed a prevalence rate of 3.01% with control group showing a 1.62% prevalence rate.

The prevalence rate of BMS observed in the present study was higher than reported by Lipton JA, Ship 0.7% (18). Bergdahl and Anneroth 0.8%, (19). but lower than that reported by Basker Hakeberg M, 4.6%. (20) Bergdahl M, Bergdahl J, 3.7% (21). Femiano F, 13% (22), Savage NW (23) and Scala A (24).

The prevalence of BMS appears to be inaccurately estimated because of inappropriate and lack of universally accepted diagnostic criteria. The lower prevalence of BMS in this study may be attributed to the wide age range of patients in both sexes. When BMS was identified only on the basis of a prolonged burning sensation of the oral mucosa, a prevalence rate of 14.8% was estimated (25). However, when diagnosis was arrived at by the use of more correct criteria, BMS prevalence fell to 0.8% (19). In present study psychological component in BMS has been clearly identified. This is supported by studies that report greater levels of depression and anxiety in patients with BMS compared to control groups.

## Conclusions

Oral health is important for patients with special needs such as psychiatric patients. The results of the present study provides an information on the distribution of oral mucosal diseases in psychiatric and general population. Oral lesions are significantly seen higher in individuals with anxiety and depression than the normal healthy individuals with sound mind and body. It is the responsibility of the oral health provider to effectively provide adequate dental treatment for people with psychiatric disabilities. Hence as an adjuvant to conventional therapy in these patients, psychiatric analysis and intervention should be considered while treating these oral diseases.
